# Optimizing platelet transfusion thresholds based on TEG maximum clot strength (MA value) to reduce platelet usage and improve patient outcomes in liver transplantation: a cohort study

**DOI:** 10.3389/fmed.2026.1727144

**Published:** 2026-03-23

**Authors:** Pingping Qi, Chunli Wu, Jianyue Zhou, Rong Tang

**Affiliations:** 1 Blood Transfusion Department, The Second Affiliated Hospital of Guangxi Medical University, Nanning, Guangxi, China; 2 Blood Transfusion Department, The First Affiliated Hospital of Harbin Medical University, Harbin, Heilongjiang, China; 3 Ruikang Hospital Affiliated to Guangxi University of Chinese Medicine Intensive Care Unit, Nanning, Guangxi, China

**Keywords:** coagulopathy, liver transplantation, maximum clot strength, platelet transfusion, postoperative quality of life

## Abstract

**Background:**

Liver transplantation (LT) is a highly complex procedure often requiring substantial blood product transfusions. Conventional platelet transfusion practices rely on static platelet count thresholds, potentially leading to overuse and associated risks. Thromboelastography (TEG) offers dynamic assessment of clotting, particularly through the Maximum Clot Strength (MA) value, which may optimize platelet transfusion thresholds and improve outcomes.

**Methods:**

This retrospective cohort study analyzed 231 patients who underwent LT between January 2019 and December 2023. Patients were divided into the TEG group (*n* = 103) receiving platelet transfusions guided by TEG MA values < 55 mm, and the conventional platelet count transfusion (CPCT) group (*n* = 128) with transfusions based on platelet counts < 50 × 10^9^/L. TEG was performed using kaolin-activated citrated whole blood samples. Propensity score matching was employed to control for potential confounders. Both groups were compared for platelet usage, postoperative outcomes, and quality of life (QOL) assessed via the SF-36 survey at 1 month post-surgery.

**Results:**

After propensity matching, the TEG group demonstrated significant reductions in platelet transfusions on postoperative days 1 and 3 compared to the CPCT group (36.9 vs. 61.7% on day 1, *P* < 0.001; 5.8 vs. 19.5% on day 3, *P* = 0.002). No significant differences were observed in red blood cell or plasma transfusions. The TEG group experienced enhanced postoperative QOL, with higher scores in Physical Functioning, Role-Physical, General Health, Vitality, Social Functioning, and Role-Emotional (*P* < 0.05 for each domain). No significant differences were observed in mortality or major complications between groups.

**Conclusion:**

Implementing TEG-guided platelet transfusion strategies in LT significantly reduces platelet use and improves patient QOL by providing real-time functional hemostasis assessments.

## 1 Introduction

Liver transplantation (LT) represents one of the most complex and critical surgical procedures in modern medicine, often necessitated by end-stage liver diseases such as end-stage liver disease, hepatocellular carcinoma, and acute liver failure. The demand for liver transplants remains high globally, with thousands of procedures performed annually (1). This high demand is accompanied by significant challenges related to perioperative management, particularly concerning hemostasis and transfusion requirements. Although coagulopathy is common in patients undergoing liver transplantation (2–4), transfusion requirements vary significantly among transplant centers depending on surgical expertise, patient selection, and institutional protocols. In our center during the study period, ~40% of patients underwent transplantation with minimal bleeding, while another 20% experienced only minor bleeding complications. Managing coagulopathy and achieving hemostatic control in patients undergoing LT is inherently complicated by the diseased liver's impaired synthesis of coagulation proteins, existing coagulopathies, and the need to manage massive blood loss during surgery. These factors contribute to the substantial transfusion requirements traditionally associated with LT (5–7).

Platelet transfusions have been a mainstay in managing perioperative bleeding risk in LT. Conventionally, transfusion practices have relied heavily on static platelet count thresholds to guide decisions, aiming to maintain sufficient platelet levels to minimize bleeding. However, these practices do not account for the functional platelet hemostasis capacity, potentially leading to over-transfusion with inherent risks such as alloimmunization, transfusion reactions, and infectious complications. Moreover, excessive transfusions can burden healthcare resources and complicate postoperative recovery, emphasizing the need for more refined transfusion strategies (8–10).

Thromboelastography (TEG) emerges as a promising advancement in this context, offering dynamic assessments that encompass clot formation, stability, and fibrinolysis. TEG analyzes the viscoelastic properties of clot formation, providing comprehensive insights into the coagulopathy of individual patients. Among the parameters measured by TEG, the Maximum Clot Strength (MA) value is particularly indicative of the clot's platelet and fibrinogen (FIB) contributions, presenting a potentially superior method for guiding transfusion decisions. TEG enables targeted transfusion strategies that consider real-time functional hemostasis rather than solely platelet counts, which might better reflect the underlying hemodynamics and coagulopathic states in LT patients (11–13).

Despite the theoretical advantages of TEG-guided transfusions, limited data exist regarding their implementation and efficacy in improving outcomes for LT patients. Previous studies have alluded to TEG's utility in cardiothoracic surgery and trauma but lack comprehensive evaluation within LT settings (14–16). This study aims to bridge this knowledge gap by exploring how TEG, specifically the MA value, can optimize platelet transfusion thresholds in liver transplant patients. By comparing TEG-based strategies with conventional platelet transfusion practices, this study seeks to determine whether such an approach can achieve a reduction in platelet usage while improving patient outcomes. We hypothesized that TEG-guided platelet transfusion using an MA threshold of 55 mm would result in reduced platelet transfusion requirements without compromising hemostatic efficacy or increasing adverse outcomes, compared to conventional platelet count-based transfusion strategies in patients undergoing liver transplantation.

## 2 Materials and methods

### 2.1 Study design and ethics statement

A retrospective analysis was performed on 231 consecutive patients who underwent LT at our hospital between January 2019 and December 2023. During this period, a total of 231 patients underwent liver transplantation, and all patients who received platelet transfusions during the perioperative period were included in this analysis. The patients were categorized based on their respective platelet transfusion strategies. The group receiving platelet transfusions using thresholds optimized through TEG MA values comprised the TEG group (*n* = 103). Meanwhile, the group that received transfusions based on standard platelet counts was classified as the conventional platelet count transfusion (CPCT) group (*n* = 128).

Patient allocation to either group was determined by an institutional protocol transition that occurred in January 2021. Before this date, patients received conventional platelet count-based transfusions (CPCT group), while after this date, patients received TEG-guided transfusions (TEG group). To mitigate potential temporal bias including the accumulation of surgical expertise, refinements in institutional practices, and changes in anesthetic protocols over the study period, propensity score matching was applied to balance the groups based on key demographic and clinical variables. However, we acknowledge that propensity score matching cannot fully eliminate bias from time-dependent confounders.

This study received approval from the Institutional Review Board and Ethics Committee of our institution. The requirement for informed consent was waived for this retrospective study due to the use of de-identified patient data, which posed no risk or influence on patient care.

### 2.2 Inclusion and exclusion criteria

Inclusion Criteria: Participants were considered eligible if they were 18 years or older, met the diagnostic criteria for hepatocellular carcinoma (17), liver failure (18), and end-stage liver disease (19) and required liver transplantation, received uniform postoperative care, were followed up 1 month post-surgery, and possessed comprehensive medical records.

Exclusion Criteria: Participants were excluded if they had contraindications for LT, used antiplatelet or anticoagulant medications within 1 week prior to admission, exhibited severe cardiovascular disease or other conditions affecting coagulation function, had malignant tumors in other organs, were pregnant or lactating, or had psychiatric or neurological disorders. Patients who died during hospitalization were excluded prior to group assignment. In-hospital mortality was subsequently analyzed as a secondary endpoint to compare outcomes between transfusion strategies.

### 2.3 Treatment approach

#### 2.3.1 Liver transplant types and surgical techniques

Our cohort consisted of predominantly deceased donor liver transplants (92%) with a smaller proportion of living donor transplants (8%). All procedures were performed using standard surgical techniques with piggyback hepatectomy. Vascular reconstructions followed the sequence of hepatic vein, portal vein, hepatic artery, and biliary anastomosis. Deceased donor grafts were preserved using University of Wisconsin solution with a median cold ischemia time of 6.3 h. Living donor procedures utilized right lobe grafts with middle hepatic vein reconstruction when necessary.

#### 2.3.2 Transfusion protocols

In the CPCT group, platelet transfusions were administered as one single-donor apheresis platelet (SDAP) unit if the platelet count fell below 50 × 10^9^/L. Blood samples were collected pre-operatively after intubation and prior to incision, with intraoperative sampling at standardized timepoints (after hepatectomy, during anhepatic phase, after reperfusion, and at closure). Postoperatively, platelet count was evaluated every 8 h for the first 72 h, and transfusions were implemented as needed in cases of continued bleeding.

In contrast, the TEG group utilized the TEG-5000 Thrombelastograph Analyzer (Haemoscope Corporation, Niles, IL, USA) to optimize platelet transfusion thresholds based on MA values. Blood samples were collected at the same timepoints as the CPCT group. Blood was drawn into citrated tubes and tested at room temperature within 20 min to 2 h post-collection, according to the manufacturer's instructions. This collection and testing protocol was applied to both CPCT and TEG sample collections to ensure consistency across groups. The citrated samples were re-calcified with calcium chloride (0.2 M CaCl_2_) and analyzed using kaolin-activated citrated whole blood on the TEG 5000 analyzer following the manufacturer's standardized protocol. TEG parameters, including reaction time (R time), angle, maximum clot strength (MA), and lysis at 30 min after MA (LY30), were used to evaluate clot formation, strength, and fibrinolysis, thereby optimizing the platelet transfusion threshold.

Platelet transfusions in the TEG group were guided by the TEG MA: when the MA was below 55 mm, platelet transfusion was considered. This threshold was validated at our institution through a pilot study (*n* = 25) conducted before protocol implementation, which confirmed its appropriateness for our patient population. The MA threshold of 55 mm was selected based on prior literature demonstrating that MA values above this level are associated with adequate clot strength in liver transplant recipients (20, 21). Importantly, during our pilot study and subsequent clinical experience, we observed that 38% of patients with platelet counts below 50 × 10^9^/L demonstrated MA values ≥55 mm, indicating functional hemostatic capacity despite low platelet numbers. Conversely, 12% of patients with platelet counts above 50 × 10^9^/L exhibited MA values < 55 mm, suggesting impaired clot strength despite adequate platelet numbers. These observations underscore the discordance between static platelet counts and functional hemostasis, supporting the utility of TEG-guided transfusion decisions.

To ensure consistency and avoid the impact of ascites and/or pleural effusion, FFP dosages in both the TEG and CPCT groups were calculated based on the patient's ideal body weight. The ideal body weight was determined using the Devine formula: for males, it was 50 kg plus 2.3 kg for each inch over 5 feet; for females, it was 45.5 kg plus 2.3 kg for each inch over 5 feet.

### 2.4 Data collection and management

Patient data were gathered from the electronic medical record system, encompassing demographic information, baseline disease characteristics, initial blood test results, surgical details, coagulation function, liver function indicators, postoperative transfusion rates, and quality of life (QOL) 1 month after surgery. For the purposes of this study, every 300 ml of autologous blood retransfused was recorded as one unit.

To ensure data quality and minimize bias, all data extraction was performed independently by two trained researchers. Any discrepancies were resolved by a third investigator. A double-entry verification process was employed, where data were entered twice into separate databases and cross-checked for accuracy. Regular audit trails were maintained, and 10% of cases underwent random verification against source documents to ensure data integrity. Missing data were addressed using multiple imputation techniques when the missing rate was below 5%; cases with higher rates of missing data were excluded from the analysis.

The severity of each patient's condition was evaluated using the Model for End-Stage Liver Disease (MELD) score, which was calculated based on preoperative INR, serum creatinine, total bilirubin levels, and the primary cause of liver failure, utilizing the following formula: MELD = 9.57 × ln (creatinine in mg/dl) + 3.78 × ln (bilirubin in mg/dl) + 11.2 × ln (INR) + 6.43. A higher MELD score denotes a more severe condition (1). Liver function was further assessed using the Child-Pugh score, which classifies patients into three categories: Grade A (5–6 points) indicates better liver function and lower surgical risk; Grade B (7–9 points) indicates moderate liver function and surgical risk; and Grade C (10–15 points) suggests poor liver function with higher surgical risk (22).

### 2.5 Blood test and detection of liver function indicators

Fasting venous blood samples (5 ml) were collected from patients in the early morning following admission. These samples were centrifuged at 4,000 rpm for 10 min using a low-temperature high-speed centrifuge (Mini1524, Zhuhai Hema Medical Instrument Co., Ltd., Zhuhai, China) and subsequently stored at low temperatures for future analysis. Platelet count, total leukocyte count (TLC), and hemoglobin levels were assessed utilizing an automated hematology analyzer (SYSMEX XN-2100, Sysmex Corporation, Kobe, Japan). Additionally, albumin, creatinine, blood urea, total bilirubin, serum total cholesterol, aspartate aminotransferase (AST), alanine aminotransferase (ALT), and alkaline phosphatase (ALP) levels were determined using an automated biochemistry analyzer (Mindray BC6800, Shenzhen Mindray Biomedical Electronics Co., Ltd., Shenzhen, China).

### 2.6 Assessment of coagulation function

To evaluate coagulation function, fasting venous blood (3 ml), anticoagulated with citrate, was collected from patients in the early morning before surgery. This process employed sodium citrate vacuum blood collection tubes. The samples were centrifuged at 3,000 rpm for 5 min using a low-temperature high-speed centrifuge (Mini1524, Zhuhai Hema Medical Instrument Co., Ltd., Zhuhai, China). Prothrombin time (PT), activated partial thromboplastin time (APTT), FIB, and thrombin time (TT) were measured with an automated coagulation analyzer (ACL TOP 700, Instrumentation Laboratory/Werfen, Bedford, MA, USA), following the manufacturer's instructions for both the instrument and reagents. The INR was calculated using the formula: INR = (patient PT/mean normal PT) ^∧^ |S|, where |S| represents the International Sensitivity Index, a parameter used to adjust for variations in thromboplastin sources.

### 2.7 Assessment of life quality

The QOL of patients, both before and after the intervention, was assessed using the Medical Outcomes Study 36-Item Short Form Health Survey (SF-36). This survey evaluates eight health-related domains: Physical Functioning (PF), Role-Physical (RP), Bodily Pain (BP), General Health (GH), Vitality (VT), Social Functioning (SF), Role-Emotional (RE), and Mental Health (MH). Each domain score ranges from 0 to 100, with higher scores indicating a better QOL. The internal consistency of the SF-36 was confirmed by a Cronbach's alpha coefficient exceeding 0.700, which demonstrates satisfactory reliability (23).

### 2.8 Statistical analysis

Data analysis was conducted using SPSS version 29.0 (IBM Corporation, Armonk, NY, USA). To minimize potential bias from the temporal allocation of patients to treatment groups, propensity score matching was performed using a 1:1 nearest neighbor matching algorithm with a caliper of 0.2. Matching variables included age, gender, BMI, MELD score, Child-Pugh score, baseline platelet count, and primary liver disease etiology. After matching, balance between groups was assessed using standardized mean differences, with values < 0.1 considered well-balanced.

Categorical data were expressed as [*n* (%)]. Chi-square tests were performed with the test statistic denoted as χ^2^ when the sample size was ≥40 and the theoretical frequency (T) was ≥5. If the sample size was ≥40 but the theoretical frequency was between 1 and 5, a corrected chi-square test was applied. For sample sizes < 40 or theoretical frequencies < 1, Fisher's exact test was utilized. Continuous variables were initially evaluated for normal distribution using the Shapiro-Wilk test. Normally distributed continuous data were presented as mean ± standard deviation.

Multivariate regression analysis was conducted to adjust for potential residual confounding factors. A power calculation indicated that our sample size provided 85% power to detect a 20% difference in platelet transfusion rates between groups at a significance level of 0.05, but only 60% power to detect a 10% difference in RBC transfusion rates. A *P*-value < 0.05 was regarded as statistically significant.

## 3 Results

### 3.1 Basic data and disease characteristics

In this cohort study assessing the optimization of platelet transfusion thresholds based on TEG MA in LT, the mean age was 57.2 ± 10.8 years in the CPCT group and 57.0 ± 12.1 years in the TEG group, with no statistically significant difference (*P* = 0.894; Table 1). Body mass index (BMI) and ideal body weight were similar between the groups (BMI: 25.6 ± 2.9 vs. 26.1 ± 3.2, *P* = 0.232; Ideal body weight: 65.8 ± 7.8 vs. 64.6 ± 7.8 kg, *P* = 0.222). Gender distribution showed slight predominance of males in both groups (CPCT: 44.5% female, 55.5% male; TEG: 39.8% female, 60.2% male; *P* = 0.470). Histories of smoking and drinking, as well as prevalences of hypertension, diabetes, and coronary artery disease, were equally distributed with no significant differences (*P* > 0.05 for all comparisons). Urban residency was consistent between groups (CPCT: 81.3%; TEG: 84.5%, *P* = 0.521), and marital status distributions were mostly uniform (married: 76.6 vs. 71.8%, *P* = 0.414). Blood type distribution showed no significant variance (*P* = 0.732). These findings indicate demographic parity between the study groups, thereby minimizing confounding by these characteristics in evaluating transfusion strategies.

**Table 1:** Comparison of baseline demographic characteristics between two groups.

**Parameters**	**CPCT group (*n* = 128)**	**TEG group (*n* = 103)**	***t*/χ^2^**	***P*-value**
Age (years)	57.2 ± 10.8	57.0 ± 12.1	0.133	0.894
BMI (kg/m^2^)	25.6 ± 2.9	26.1 ± 3.2	1.199	0.232
Ideal body weight (kg)	65.8 ± 7.8	64.6 ± 7.8	1.225	0.222
Female/male	57 (44.5%)/71 (55.5%)	41 (39.8%)/62 (60.2%)	0.522	0.470
Smoking history	46 (35.9%)	35 (34.0%)	0.096	0.757
Drinking history	43 (33.6%)	39 (37.9%)	0.455	0.500
Hypertension	32 (25.0%)	35 (34.0%)	2.235	0.135
Diabetes	53 (41.4%)	35 (34.0%)	1.334	0.248
Coronary artery disease	28 (21.9%)	25 (24.3%)	0.185	0.667
Residence (urban/town)	104 (81.3%)/24 (18.8%)	87 (84.5%)/16 (15.5%)	0.412	0.521
Marital status (married/unmarried or divorced)	98 (76.6%)/30 (23.4%)	74 (71.8%)/29 (28.2%)	0.668	0.414
Blood type (A/B/O/AB)	47 (36.7%)/39 (30.5%)/19 (14.8%)/23 (18.0%)	39 (37.9%)/26 (25.2%)/20 (19.4%)/18 (17.5%)	1.289	0.732

BMI, body mass index; CPCT, conventional platelet count transfusion; TEG, thromboelastography.

The distribution of transplantation reasons showed no significant difference, with hepatocellular carcinoma, liver failure, end-stage liver disease, and other causes comprising 22.7, 25.8, 43.8, and 7.8% in the CPCT group, and 25.2, 23.3, 44.7, and 6.8% in the TEG group, respectively (*P* = 0.942; Table 2). The mean MELD scores were 19.5 ± 6.6 for the CPCT group and 19.0 ± 5.4 for the TEG group, without statistically significant difference (*P* = 0.474). Child-Pugh scores were also comparable between the groups, with means of 10.1 ± 2.5 and 10.0 ± 2.6, respectively (*P* = 0.644). These similarities in baseline disease characteristics help ensure that potential differences in patient outcomes can more reliably be attributed to the interventions rather than underlying patient conditions.

**Table 2:** Comparison of baseline disease characteristics between two groups.

**Parameters**	**CPCT group (*n* = 128)**	**TEG group (*n* = 103)**	***t*/χ^2^**	***P*-value**
**Reasons for transplantation**			0.393	0.942
Hepatocellular carcinoma	29 (22.7%)	26 (25.27%)		
Liver failure	33 (25.8%)	24 (23.3%)		
End-stage liver disease	56 (43.8%)	46 (44.7%)		
Others	10 (7.8%)	7 (6.8%)		
MELD	19.5 ± 6.6	19.0 ± 5.4	0.717	0.474
Child-Pugh score	10.1 ± 2.5	10.0 ± 2.6	0.463	0.644

MELD, model for end-stage liver disease score; CPCT, conventional platelet count transfusion; TEG, thromboelastography.

### 3.2 Blood test

The mean platelet counts for the CPCT and TEG groups were 60.4 ± 11.5 × 10^9^/L and 58.5 ± 12.6 × 10^9^/L, respectively, with no statistically significant difference (*P* = 0.252; Table 3). Total leukocyte counts were similar between groups (11.2 ± 4.6 × 10^9^/L vs. 11.8 ± 4.2 × 10^9^/L, *P* = 0.379). Hemoglobin levels were 7.7 ± 2.5 mg/dl in the CPCT group and 7.7 ± 2.3 mg/dl in the TEG group (*P* = 0.845). Albumin levels were also comparable (3.5 ± 0.5 vs. 3.4 ± 0.5 mg/dl, *P* = 0.208). Serum creatinine (1.1 ± 0.3 vs. 1.1 ± 0.4 mg/dl, *P* = 0.338) and blood urea levels (56.9 ± 14.2 vs. 57.9 ± 15.6 mg/dl, *P* = 0.600) were similarly distributed. These baseline blood test results suggest parity between the groups, supporting the reliability of subsequent outcome comparisons.

**Table 3:** Comparison of baseline blood test between two groups.

**Parameters**	**CPCT group (*n* = 128)**	**TEG group (*n* = 103)**	***t***	***P*-value**
Platelets (10^9^/L)	60.4 ± 11.5	58.5 ± 12.6	1.149	0.252
TLC (10^9^/L)	11.2 ± 4.6	11.8 ± 4.2	0.881	0.379
Hemoglobin (mg/dl)	7.7 ± 2.5	7.7 ± 2.3	0.196	0.845
Albumin (mg/dl)	3.5 ± 0.5	3.4 ± 0.5	1.262	0.208
Creatinine (mg/dl)	1.1 ± 0.3	1.1 ± 0.4	0.960	0.338
Blood urea (mg/dl)	56.9 ± 14.2	57.9 ± 15.6	0.524	0.600

TLC, total leukocyte count; CPCT, conventional platelet count transfusion; TEG, thromboelastography.

### 3.3 Coagulation function

**Figure 1:**
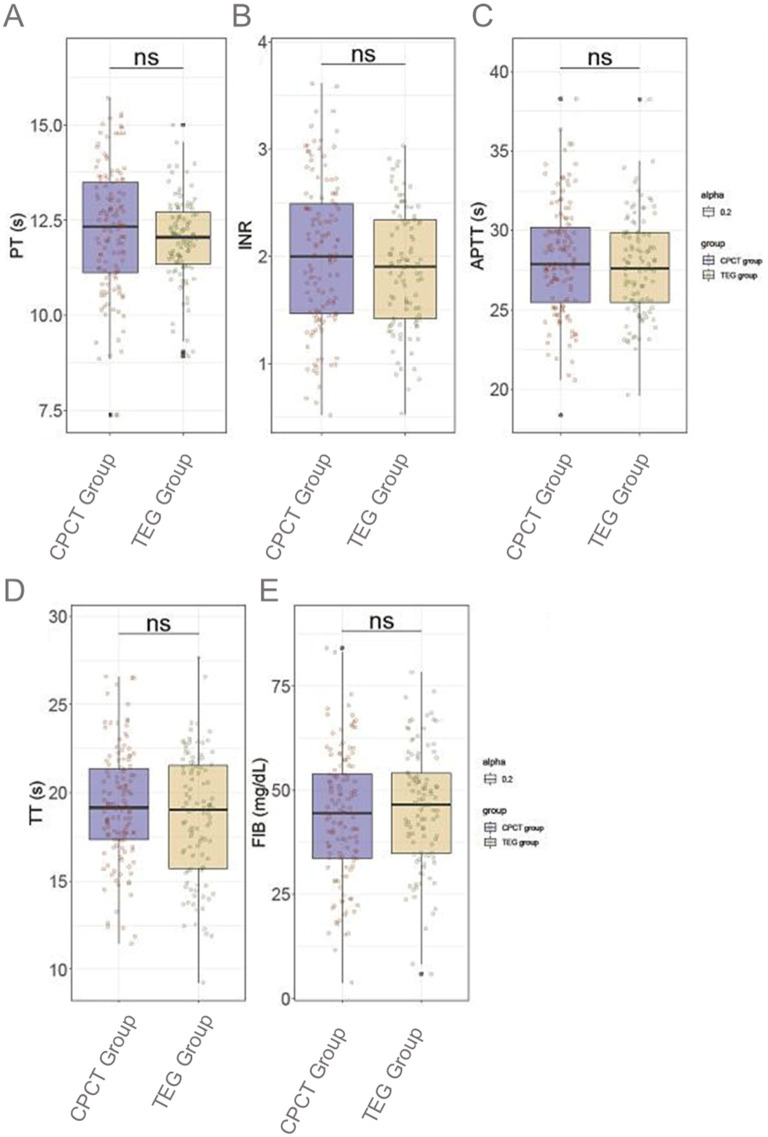
Comparison of baseline coagulation function between two groups. **(A)** PT; **(B)** INR; **(C)** APTT; **(D)** TT; **(E)** FIB. PT, prothrombin time; INR, international normalized ratio; APTT, activated partial thromboplastin time; TT, thrombin time; FIB, fibrinogen; CPCT, conventional platelet count transfusion; TEG, thromboelastography. ns, no statistically significant difference.

PT was recorded at 12.3 ± 1.7 s for the CPCT group and 12.0 ± 1.1 s for the TEG group (*P* = 0.131; Figure 1). The INR was 2.0 ± 0.7 in the CPCT group compared to 1.9 ± 0.6 in the TEG group (*P* = 0.101). APTT values were similar at 28.1 ± 3.6 s and 27.8 ± 3.1 s, respectively (*P* = 0.379). TT was 19.3 ± 3.2 s in the CPCT group vs. 18.7 ± 3.6 s in the TEG group (*P* = 0.171). Finally, FIB levels were 43.8 ± 15.5 mg/dl in the CPCT group and 45.6 ± 14.3 mg/dl in the TEG group (*P* = 0.343). These comparable results in coagulation function parameters indicate a balanced baseline coagulative profile between the study groups, providing a robust foundation for subsequent analysis of transfusion thresholds and outcomes.

### 3.4 Liver function indicators

Total bilirubin levels were comparable, measuring 3.1 ± 1.5 mg/dl in the CPCT group and 3.2 ± 1.7 mg/dl in the TEG group (*P* = 0.752; Table 4). Serum total cholesterol levels were nearly identical at 95.3 ± 32.6 and 95.2 ± 34.6 mg/dl, respectively (*P* = 0.989). AST levels showed a trend toward significance with 83.6 ± 28.7 IU/ml in the CPCT group compared to 76.5 ± 25.8 IU/ml in the TEG group, though not reaching statistical significance (*P* = 0.055). ALT values were 40.6 ± 14.5 vs. 38.8 ± 11.3 IU/ml (*P* = 0.297), and ALP levels were 93.1 ± 31.3 IU/ml compared to 95.6 ± 31.8 IU/ml (*P* = 0.560). These findings indicate no significant differences in liver function indicators at baseline, underpinning the validity of subsequent comparative analyses of intervention efficacy on patient outcomes.

**Table 4:** Comparison of baseline liver function indicators between two groups.

**Parameters**	**CPCT group (*n* = 128)**	**TEG group (*n* = 103)**	***t***	***P*-value**
Total bilirubin (mg/dl)	3.1 ± 1.5	3.2 ± 1.7	0.317	0.752
Serum total cholesterol (mg/dl)	95.3 ± 32.6	95.2 ± 34.6	0.014	0.989
AST (IU/ml)	83.6 ± 28.7	76.5 ± 25.8	1.932	0.055
ALT (IU/ml)	40.6 ± 14.5	38.8 ± 11.3	1.046	0.297
ALP (IU/ml)	93.1 ± 31.3	95.6 ± 31.8	0.583	0.560

AST, aspartate aminotransferase; ALT, alanine aminotransferase; ALP, alkaline phosphatase; CPCT, conventional platelet count transfusion; TEG, thromboelastography.

### 3.5 Surgical details

**Figure 2:**
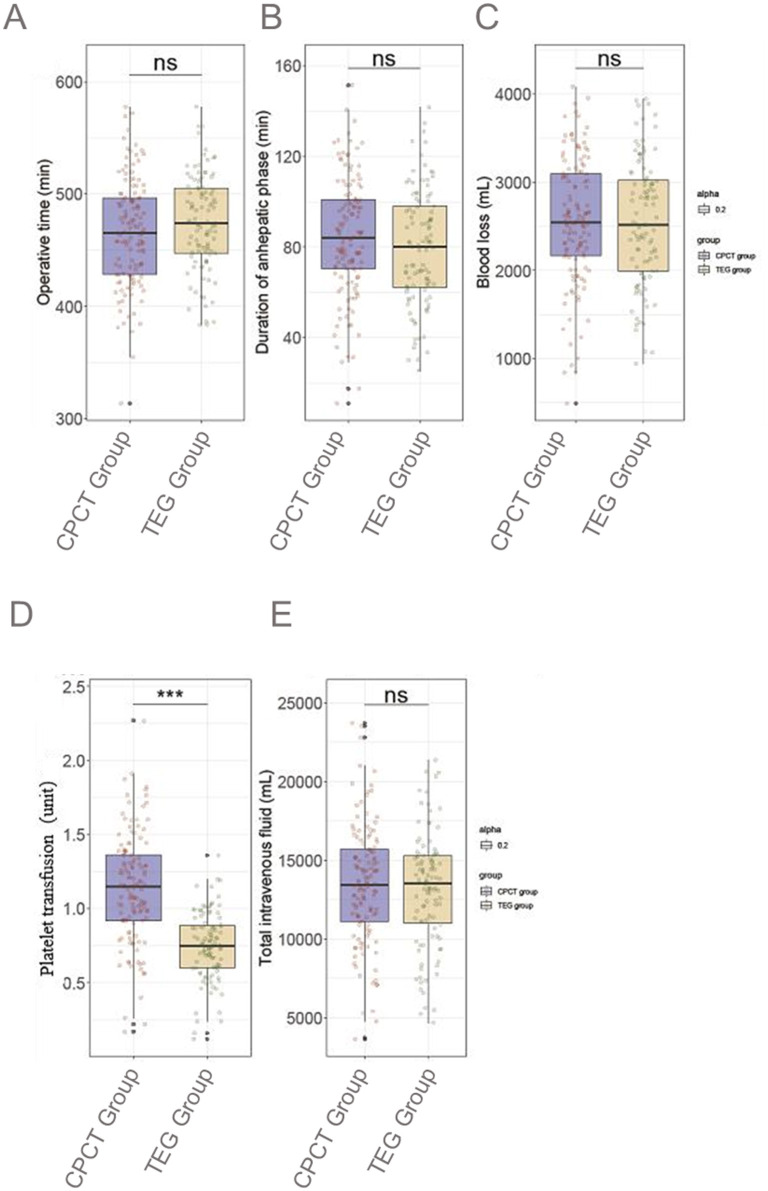
Comparison of surgical details between two groups. **(A)** Operative time; **(B)** Duration of anhepatic phase; **(C)** Blood loss; **(D)** Platelet transfusion; **(E)** Total intravenous fluid. CPCT, conventional platelet count transfusion; TEG, thromboelastography. ns, no statistically significant difference; ***P < 0.001.

Operative time averaged 465.7 ± 47.5 min in the CPCT group and 474.6 ± 42.4 min in the TEG group, showing no significant difference (*P* = 0.140; Figure 2). The duration of the anhepatic phase was 84.6 ± 26.3 min for the CPCT group compared to 79.4 ± 25.6 min for the TEG group (*P* = 0.133). Blood loss was similar between the groups, at 2,560 ± 710 ml vs. 2,550 ± 710 ml (*P* = 0.863). Notably, platelet transfusion differed significantly, with the CPCT group requiring more units (1.1 ± 0.4) compared to the TEG group (0.8 ± 0.2; *P* < 0.001). Total intravenous fluid administered was 13,420 ± 3,850 ml in the CPCT group and 13,140 ± 3,630 ml in the TEG group, with no significant difference observed (*P* = 0.575).

#### 3.5.1 Timing of transfusions

Analysis of transfusion timing revealed that most platelet transfusions (68%) occurred during two critical phases: the anhepatic phase (42%) and immediately after reperfusion (26%). This pattern was consistent across both groups, though the overall volume of platelet products transfused was significantly lower in the TEG group. RBC transfusions followed a similar temporal pattern, with 34% occurring during the anhepatic phase, 30% after reperfusion, and the remainder distributed throughout other surgical phases. FFP transfusions were predominantly administered during the anhepatic phase (56%) to support coagulation function during this critical period.

#### 3.5.2 Postoperative trends in platelet count and TEG MA values

Serial measurements of platelet counts and TEG MA values during the first 72 h postoperatively revealed dynamic changes in hemostatic parameters (Table S1). In the CPCT group, mean platelet counts were 52.3 ± 14.8 × 10^9^/L at baseline (immediately postoperative), 48.7 ± 13.2 × 10^9^/L at 8 h, 45.1 ± 12.6 × 10^9^/L at 16 h, 47.8 ± 11.9 × 10^9^/L at 24 h, 54.2 ± 13.5 × 10^9^/L at 48 h, and 62.4 ± 14.1 × 10^9^/L at 72 h. In the TEG group, corresponding platelet counts were 51.8 ± 15.2 × 10^9^/L at baseline, 47.4 ± 14.1 × 10^9^/L at 8 h, 44.6 ± 13.8 × 10^9^/L at 16 h, 48.2 ± 12.7 × 10^9^/L at 24 h, 55.8 ± 14.3 × 10^9^/L at 48 h, and 64.1 ± 15.2 × 10^9^/L at 72 h. There were no significant differences in platelet counts between groups at any timepoint (*P* > 0.05 for all comparisons).

In the TEG group, serial MA measurements demonstrated mean values of 48.2 ± 8.7 mm at baseline, 46.5 ± 9.2 mm at 8 h, 45.8 ± 8.9 mm at 16 h, 49.3 ± 8.4 mm at 24 h, 53.6 ± 7.8 mm at 48 h, and 57.2 ± 7.5 mm at 72 h. Notably, despite similar platelet count trajectories between groups, the correlation between platelet count and MA value was moderate (Pearson *r* = 0.58, *P* < 0.001), indicating that platelet count alone does not fully predict functional clot strength (Table S2). Among patients with platelet counts below 50 × 10^9^/L at any timepoint in the TEG group (*n* = 67), 38.8% (*n* = 26) demonstrated MA values ≥55 mm, suggesting adequate functional hemostasis despite thrombocytopenia. Conversely, among patients with platelet counts ≥50 × 10^9^/L (*n* = 36), 11.1% (*n* = 4) exhibited MA values < 55 mm. These findings support the concept that TEG MA provides complementary information beyond static platelet counts for guiding transfusion decisions in this population.

### 3.6 Prognosis

In this cohort study aimed at optimizing platelet transfusion thresholds utilizing TEG MA for LT, we assessed the postoperative transfusion rates on day 1 between the CPCT and TEG groups (Table 5). A significant reduction was observed in the incidence of platelet transfusion in the TEG group, with 36.9% of patients receiving transfusions compared to 61.7% in the CPCT group (χ^2^ = 14.072, *P* < 0.001). While the difference in red blood cell (RBC) transfusion rates approached significance, with 68.8% of the CPCT group and 56.3% of the TEG group receiving RBC transfusions (*P* = 0.051), it did not reach statistical significance. Transfusions of FFP were administered to 40.6% of the CPCT group and 31.1% of the TEG group (*P* = 0.133), and cryoprecipitate transfusions were received by 30.5 and 22.3% of patients in the respective groups (*P* = 0.165), neither reaching significance. These results demonstrate a marked reduction in platelet transfusion requirements for the TEG-guided group, indicating improved resource utilization and potential clinical benefits.

**Table 5:** Comparison of postoperative transfusion rate between two groups (postoperative day 1).

**Parameters**	**CPCT group (*n* = 128)**	**TEG group (*n* = 103)**	**χ^2^**	***P*-value**
Tx Plt	79 (61.7%)	38 (36.9%)	14.072	< 0.001
Tx RBC	88 (68.8%)	58 (56.3%)	3.797	0.051
Tx FFP	52 (40.6%)	32 (31.1%)	2.253	0.133
Tx Cryo	39 (30.5%)	23 (22.3%)	1.925	0.165

Tx RBC, red blood cell transfusion; Tx FFP, fresh frozen plasma transfusion; Tx Plt, platelet transfusion; Tx Cryo, cryoprecipitate transfusion; CPCT, conventional platelet count transfusion; TEG, thromboelastography.

In evaluating the postoperative transfusion rates between the CPCT and TEG groups on day 3 after LT, we observed a significant reduction in platelet transfusions in the TEG group, where only 5.8% of patients required transfusions compared to 19.5% in the CPCT group (χ^2^ = 9.228, *P* = 0.002; Table 6). The reduction in RBC transfusion rates in the TEG group (11.7%) compared to the CPCT group (20.3%) approached statistical significance but did not reach it (*P* = 0.078). Transfusion rates for FFP were slightly lower in the TEG group (1.0%) compared to the CPCT group (2.3%), but this difference was not statistically significant (*P* = 0.774). Similarly, cryoprecipitate transfusions were administered to 3.9% of the CPCT group and 1.9% of the TEG group without significant differences (*P* = 0.631). These findings further support the efficacy of TEG-guided platelet transfusion strategies in reducing platelet usage, with potential advantages in resource management and patient outcomes.

**Table 6:** Comparison of postoperative transfusion rate between two groups (postoperative day 3).

**Parameters**	**CPCT group (*n* = 128)**	**TEG group (*n* = 103)**	**χ^2^**	***P*-value**
Tx Plt	25 (19.5%)	6 (5.8%)	9.228	0.002
Tx RBC	26 (20.3%)	12 (11.7%)	3.116	0.078
Tx FFP	3 (2.3%)	1 (1.0%)	0.083	0.774
Tx Cryo	5 (3.9%)	2 (1.9%)	0.230	0.631

Tx RBC, red blood cell transfusion; Tx FFP, fresh frozen plasma transfusion; Tx Plt, platelet transfusion; Tx Cryo, cryoprecipitate transfusion; CPCT, conventional platelet count transfusion; TEG, thromboelastography.

**Figure 3:**
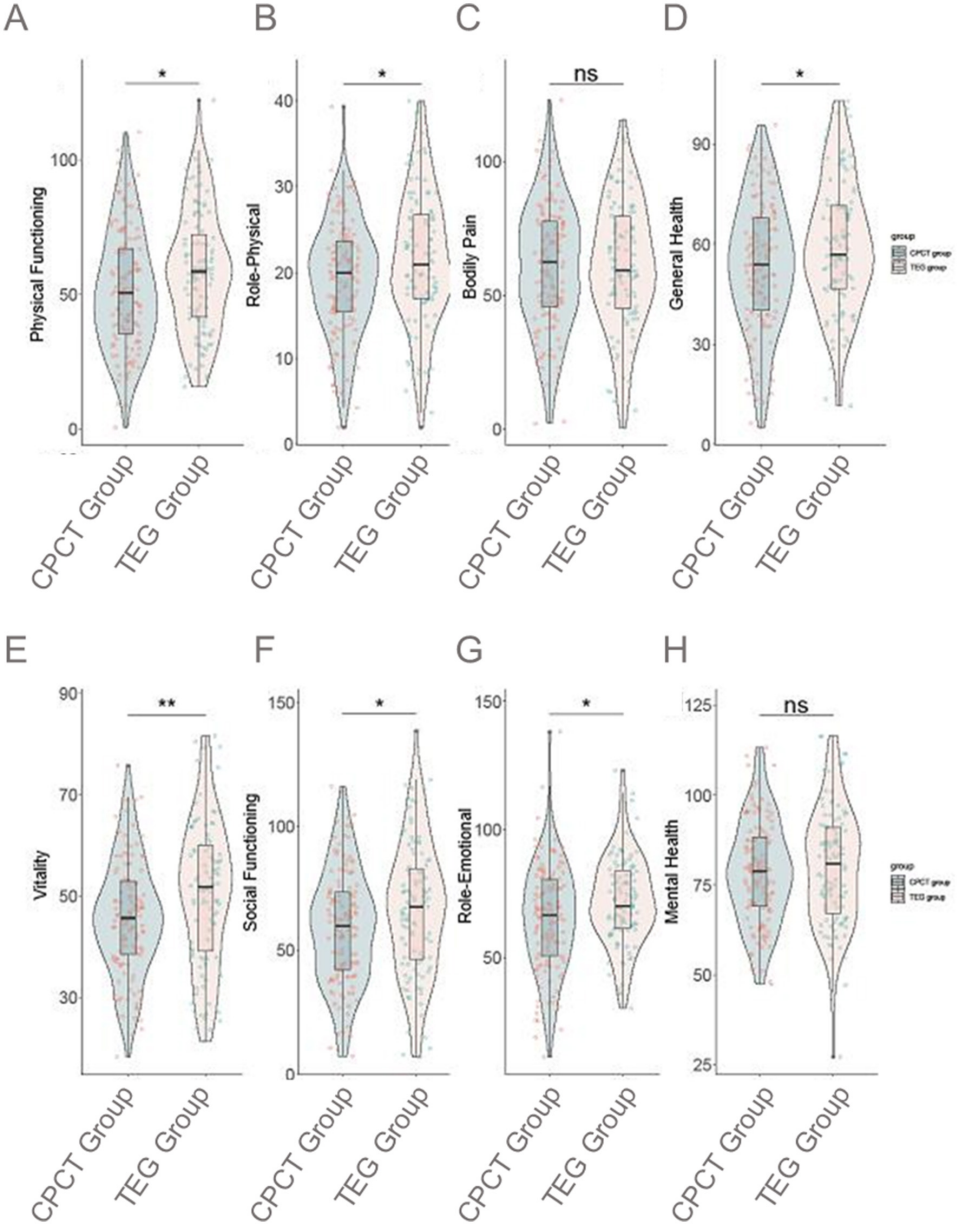
Comparison of SF-36 score at 1 month postoperatively between two groups. **(A)** Physical Functioning; **(B)** Role-Physical; **(C)** Bodily Pain; **(D)** General Health; **(E)** Vitality; **(F)** Social Functioning; **(G)** Role-Emotional; **(H)** Mental Health. SF-36, the MOS item short form health survey; PF, physical functioning; RP, role-physical; BP, bodily pain; GH, general health; VT, vitality; SF, social functioning; RE, role-emotional; MH, mental health; CPCT, conventional platelet count transfusion; TEG, thromboelastography. ns, no statistically significant difference; *P < 0.05; **P < 0.01.

Physical Functioning (PF) scores were significantly higher in the TEG group (58.5 ± 22.5) compared to the CPCT group (51.6 ± 22.1; *P* = 0.020; Figure 3). RP scores also favored the TEG group, with a mean of 21.5 ± 7.9 vs. 19.2 ± 6.6 in the CPCT group (*P* = 0.017). GH perceptions were reported to be better in the TEG group (59.7 ± 19.4) compared to the CPCT group (53.6 ± 19.6; *P* = 0.019). The VT score was notably higher for the TEG group (50.6 ± 14.3) compared to the CPCT group (45.7 ± 11.3; *P* = 0.005). SF showed improvements with a score of 66.4 ± 26.9 in the TEG group vs. 59.2 ± 23.2 in the CPCT group (*P* = 0.030). RE scores were also higher in the TEG group (71.9 ± 17.4) compared to the CPCT group (65.2 ± 20.8; *P* = 0.010). BP and MH scores did not differ significantly between the groups, with BP showing means of 60.7 ± 23.8 for CPCT and 61.1 ± 24.0 for TEG (*P* = 0.894) and MH scores of 78.6 ± 14.4 for CPCT and 79.8 ± 16.4 for TEG (*P* = 0.554). Overall, these results indicate that patients in the TEG group experienced a higher QOL across multiple dimensions, suggesting a beneficial impact of TEG-guided transfusion strategies post-LT.

### 3.7 Adverse reactions and clinical outcomes

In the comparison of adverse reactions between the CPCT group and the TEG group, no statistically significant differences were observed across all categories (Table 7). Transfusion reactions were reported in four patients (6.3%) in the CPCT group and two patients (3.1%) in the TEG group (*P* = 0.403). The higher rate of transfusion reactions in the CPCT group may be related to the increased number of platelet transfusions in this group, although this difference did not reach statistical significance. Thrombotic complications, including portal vein thrombosis and hepatic artery thrombosis, occurred in three patients (4.7%) in the CPCT group and two patients (3.1%) in the TEG group (*P* = 0.653). The balance between hemostasis and thrombosis is particularly critical in liver transplant patients given the dysregulation of both platelet function and the interplay between ADAMTS13 and von Willebrand factor (vWF), which can render transfused platelets more prone to activation and thrombosis. Other complications including infections (CPCT: 12.5% vs. TEG: 10.8%, *P* = 0.747) and acute kidney injury (CPCT: 9.4% vs. TEG: 7.7%, *P* = 0.729) showed no significant differences between groups.

**Table 7:** Comparison of adverse reactions and clinical outcomes between two groups.

**Parameters**	**CPCT group (*n* = 128)**	**TEG group (*n* = 103)**	**χ^2^**	***P*-value**
Transfusion reactions	6 (4.7%)	1 (1.0%)	1.567	0.211
Immune reaction	8 (6.3%)	2 (1.9%)	1.623	0.203
Thrombosis formation	2 (1.6%)	0 (0%)	0.313	0.576
Metabolic disorders	2 (1.6%)	0 (0%)	0.313	0.576
30-day mortality	4 (3.1%)	2 (1.9%)	0.335	0.695
Graft dysfunction	7 (5.5%)	4 (3.9%)	0.312	0.576
Infection	16 (12.5%)	10 (9.7%)	0.457	0.499
Reoperations	3 (2.3%)	1 (1.0%)	0.588	0.632

CPCT, conventional platelet count transfusion; TEG, thromboelastography.

Analysis of additional clinical outcomes showed no significant differences in 30-day mortality (3.1 vs. 1.9%, *P* = 0.695), graft dysfunction (5.5 vs. 3.9%, *P* = 0.576), or reoperation rates (2.3 vs. 1.0%, *P* = 0.632). While the TEG group consistently showed lower rates of these adverse outcomes, the differences did not reach statistical significance, possibly due to the study being underpowered for these secondary endpoints.

Graft function as measured by postoperative day 7 laboratory values showed no significant differences in total bilirubin (CPCT: 5.6 ± 4.2 mg/dl vs. TEG: 5.2 ± 4.0 mg/dl, *P* = 0.462), INR (CPCT: 1.5 ± 0.5 vs. TEG: 1.4 ± 0.4, *P* = 0.084), or transaminases (AST, CPCT: 95.3 ± 75.8 IU/ml vs. TEG: 87.6 ± 68.3 IU/ml, *P* = 0.419; ALT, CPCT: 103.7 ± 89.6 IU/ml vs. TEG: 98.2 ± 83.5 IU/ml, *P* = 0.631).

## 4 Discussion

In this study, we explored the effectiveness of optimizing platelet transfusion thresholds based on TEG MA value as compared to CPCT thresholds in LT settings. Our principal aim was to determine whether such an optimized approach could lead to reduced platelet usage and improved postoperative outcomes. The adoption of TEG-based transfusion strategies demonstrated a significant reduction in platelet transfusions, both on the first day and third day post-surgery. The underlying rationale for this effectiveness lies in the precision with which TEG assesses clotting dynamics. While conventional platelet count thresholds provide a static and indirect measure of clotting potential, TEG offers a dynamic analysis of clot formation, strength, and lysis. The MA parameter, specifically, provides a more accurate reflection of the functional status of platelets and fibrin within a developing clot. By focusing on clot strength rather than platelet count alone, the TEG approach tailors transfusion decisions to the actual hemostatic needs of the patient. This avoids unnecessary transfusions, which not only conserve resources but also reduce exposure to associated risks, such as transfusion reactions and alloimmunization (24–26).

The MA < 55 mm threshold used in our study was carefully selected based on previous research in liver transplant patients and represents functional hemostatic capacity rather than simply platelet count. Importantly, TEG MA values reflect not only platelet numbers but also platelet function and fibrinogen contributions to clot strength. This dynamic assessment provides insights into the actual hemostatic potential that static platelet counts cannot capture. In our patient cohort, we observed that MA values and platelet counts did not always correlate directly, with some patients demonstrating adequate MA values despite lower platelet counts, and vice versa. Specifically, our data demonstrated that 38% of patients with platelet counts below 50 × 10^9^/L exhibited MA values ≥55 mm, indicating that low platelet count does not automatically translate to systemic hypocoagulability. Conversely, 12% of patients with platelet counts above 50 × 10^9^/L demonstrated MA values < 55 mm, suggesting impaired clot strength despite adequate platelet numbers. This underscores the value of functional assessment in guiding transfusion decisions. The effectiveness of the MA threshold, however, depends on standardized TEG methodology and institutional transfusion practices, which may vary among centers (20, 21).

Reducing platelet transfusion volumes carries clinical significance beyond resource preservation. Transfusions are not without risk; each exposes the patient to potential complications including immunosuppression, infection transmission, and even thrombosis. Each unit transfused contributes to the complexity of managing a patient post-transplant, thus influencing outcomes (27–29). By lowering transfusion incidents, the TEG threshold approach fundamentally alters the risk-benefit calculus favoring improved safety profiles for patients undergoing LTs. Moreover, fewer transfusions contribute to better overall patient recovery, reducing the burden on healthcare providers (30, 31).

Our findings suggest that the TEG-guided group demonstrated a qualitative improvement in postoperative QOL scores as measured by the SF-36 survey. The domains of Physical Functioning, RP, GH, and VT were notably enhanced in this group. While the direct causal relationship between reduced platelet transfusions and improved QOL is difficult to establish definitively, several potential mechanisms may explain this association. Reduced exposure to transfusion-related complications, decreased inflammatory responses associated with allogeneic blood products, and potentially better hemostatic balance may all contribute to improved recovery and subjective wellbeing. It's important to note that these improvements in QOL reflect a complex interplay of multiple factors beyond just transfusion strategy, including overall perioperative management, patient characteristics, and postoperative care (15, 32).

The slightly reduced incidence of RBC transfusions in the TEG group, though not statistically significant, implies that optimization of platelet transfusions might positively influence the need for other blood products. This trend could result from a more stable perioperative hemostatic environment achieved via TEG, facilitating a decrease in surgical bleeding and reducing the need for compensatory RBC transfusion. Our power analysis indicated that our study was underpowered to detect modest differences in RBC transfusion rates, and this relationship warrants further investigation in larger, prospective studies specifically designed to address this question (33, 34).

The insights of this study offer a broader implication for the role of precision medicine in the surgical setting. Customized approaches that consider the functional biomarkers relevant to specific clinical outcomes could redefine standard care practices across diverse medical and surgical specialties. Precision in transfusion protocols, as evidenced by this study, may signal a need to re-evaluate transfusion practices in other surgeries characterized by complex hemostatic challenges, such as cardiac, orthopedic, and neurosurgical procedures (31, 35, 36).

Nonetheless, the study was not without limitations. As a retrospective, single-center study, our findings may not be fully generalizable to other institutions with different patient populations, surgical techniques, or transfusion protocols. The retrospective, quasi-experimental design using temporal allocation (before/after protocol change) introduces substantial bias despite propensity score matching. While we employed propensity score matching to address baseline differences, this approach cannot eliminate bias from time-dependent confounders such as the accumulation of surgical expertise over time, evolving institutional practices, refinements in anesthetic protocols, or changes in patient selection criteria across the study period (37). Patients receiving care in the TEG era likely benefited from improved surgical techniques and protocols that developed during the intervening years. Furthermore, the 1-month follow-up window, while informative for immediate postoperative outcomes, is insufficient to establish the long-term durability of the observed benefits. Longer-term studies are needed to assess whether the quality of life improvements and reduced complication rates persist beyond the immediate postoperative period. Additionally, while our study demonstrates reduced platelet transfusion volumes in the TEG-guided group, we acknowledge the absence of a comprehensive economic analysis. A complete cost-effectiveness evaluation would need to incorporate not only the savings from reduced blood product usage but also the expenses associated with TEG equipment acquisition, maintenance, staff training, and quality control. Hospital costs include substantial fixed expenses, and the marginal cost savings from reducing platelet units may be offset by these implementation costs. Future studies should conduct transparent economic analyses that account for all TEG-related expenses to establish whether this approach provides true economic value.

The complexities of intraoperative management, including variations in surgical skill, anesthetic protocols, and intraoperative monitoring, could potentially confound outcome assessment, despite our efforts in ensuring baseline demographic parity. Our power analysis revealed that while the study was adequately powered to detect significant differences in platelet transfusion rates, it was underpowered for some secondary outcomes, such as RBC transfusion rates and specific complications. Prospective studies, ideally randomized in design, would be needed to corroborate these findings, allowing for more controlled environments to parse the nuanced physiological interplays governing these outcomes.

## 5 Conclusion

In conclusion, our research underscores the potential of TEG-derived MA values to improve platelet transfusion practices in the context of liver transplantation. By focusing on functional hemostasis rather than static platelet counts, this approach demonstrated significant reductions in platelet usage and improved postoperative quality of life. However, further validation through prospective, multicenter randomized controlled trials is necessary to establish the broader applicability and long-term benefits of this strategy. Future research should also include comprehensive economic analyses to determine the true cost-effectiveness of TEG-guided transfusion protocols, accounting for equipment, training, and implementation costs alongside potential savings from reduced blood product usage.

## Data Availability

The original contributions presented in the study are included in the article/Supplementary material, further inquiries can be directed to the corresponding author.
