# Supplementary material for: Optimizing platelet transfusion thresholds based on TEG maximum clot strength (MA value) to reduce platelet usage and improve patient outcomes in liver transplantation: a cohort study
Source: Front Med (Lausanne). 2026 Mar 23;13:1727144. doi: 10.3389/fmed.2026.1727144 (PMC13050902; doi:10.3389/fmed.2026.1727144)
Supplement: Supplementary file 1 [file Table_1.docx]

# **Supplementary Materials**

**Table S1: Serial platelet counts and TEG MA values during the first 72 hours postoperatively.**

| **Timepoint** | **CPCT Platelet Count (×10⁹/L)** | **TEG Platelet Count (×10⁹/L)** | **TEG MA (mm)** | **P (Plt)** | **P (MA)** |
| --- | --- | --- | --- | --- | --- |
| Baseline (POD 0) | 52.3 ± 14.8 | 51.8 ± 15.2 | 48.2 ± 8.7 | 0.812 | - |
| 8 hours | 48.7 ± 13.2 | 47.4 ± 14.1 | 46.5 ± 9.2 | 0.476 | - |
| 16 hours | 45.1 ± 12.6 | 44.6 ± 13.8 | 45.8 ± 8.9 | 0.781 | - |
| 24 hours (POD 1) | 47.8 ± 11.9 | 48.2 ± 12.7 | 49.3 ± 8.4 | 0.816 | - |
| 48 hours (POD 2) | 54.2 ± 13.5 | 55.8 ± 14.3 | 53.6 ± 7.8 | 0.401 | - |
| 72 hours (POD 3) | 62.4 ± 14.1 | 64.1 ± 15.2 | 57.2 ± 7.5 | 0.381 | - |

*Plt: platelet count; MA: maximum amplitude; CPCT: conventional platelet count transfusion; TEG: thromboelastography; POD: postoperative day. Data are presented as mean ± standard deviation. P values compare platelet counts between CPCT and TEG groups at each timepoint. MA values were only measured in the TEG group.*
