# Supplementary material for: Optimizing platelet transfusion thresholds based on TEG maximum clot strength (MA value) to reduce platelet usage and improve patient outcomes in liver transplantation: a cohort study
Source: Front Med (Lausanne). 2026 Mar 23;13:1727144. doi: 10.3389/fmed.2026.1727144 (PMC13050902; doi:10.3389/fmed.2026.1727144)
Supplement: Supplementary file 2 [file Table_2.docx]

**Table S2: Correlation between platelet count and TEG MA values in the TEG group.**

| **Platelet Count Category** | **n** | **MA ≥55 mm** | **MA <55 mm** |
| --- | --- | --- | --- |
| <50 × 10⁹/L | 67 | 26 (38.8%) | 41 (61.2%) |
| ≥50 × 10⁹/L | 36 | 32 (88.9%) | 4 (11.1%) |

*MA: maximum amplitude; TEG: thromboelastography. Data demonstrate the discordance between platelet count and functional clot strength as measured by TEG MA. Pearson correlation coefficient between platelet count and MA value: r = 0.58, P < 0.001.*
